# Sensitivity of Anatase and Rutile Phases of TiO_2_ to ion irradiation: Examination of the applicability of Coulomb Explosion and Thermal Spike Models

**DOI:** 10.1038/s41598-018-30281-4

**Published:** 2018-08-06

**Authors:** Haripriya Rath, B. N. Dash, A. Benyagoub, N. C. Mishra

**Affiliations:** 1 Department of Physics, Utkal University, Bhubaneswar, 751004 Odisha India; 2 Institute of Physics, Sachivalaya Marg, Bhubaneswar, 751005 Odisha India; 3 Department of Physics, Salipur College, Salipur, 754103 Odisha India; 4 CIMAP (ex-CIRIL-GANIL), CEA-CNRS-ENSICAEN-Université de Caen, F-14070 Caen Cedex, France

## Abstract

Sensitivity of the anatase and rutile phases of titanium dioxide to Swift Heavy Ion (SHI) irradiation was experimentally probed and compared with the predictions of the Coulomb explosion, analytical and inelastic thermal spike models of ion-matter interaction. Conforming to the predictions of all these models, our study indicated higher sensitivity of anatase to these ions than the rutile phase. A detailed examination however revealed that Coulomb explosion model cannot explain either the nature of variation of the interaction cross section of SHI with the energy deposited by these ions, S_e_ to the target electrons, or the relative values of the threshold electronic energy loss, S_eth_ of anatase and rutile. The analytical thermal spike (a-TS) model, using the available physicochemical data for this oxide, predicted that tracks cannot form either in anatase or in rutile by 297 MeV and 511 MeV Ni ions, while inelastic thermal spike (i-TS) model predicted formation of ion tracks by 297 MeV Ni ions and their absence with 511 MeV Ni ions in both anatase and rutile. Our observation agreed with the predictions of i-TS model albeit with a difference in the radius of the tracks. In addition, we observed halo of defect ridden crystalline region of much larger radius around the ion track. Interestingly, the radius of the halo scales with the velocity of the ions, which is opposite to the conventionally observed velocity effect.

## Introduction

An energetic ion traversing a solid loses energy mainly through two nearly independent processes: elastic collisions with the target nuclei, and electronic excitation and ionization of the target atoms. The first process called the nuclear energy loss, S_n_ dominates at low ion energies (keV/amu) and leads to direct atomic displacements. The second process called the electronic energy loss, S_e_ prevails for swift heavy ions (SHI) at high energies (MeV/amu) and creates significant atomic rearrangements in a columnar region known as latent track in various types of materials when S_e_ exceeds a threshold value S_eth_^1^. Understanding of latent tracks arising due to the transfer of energy initially acquired by the electrons to the lattice atoms have been attempted by such mechanisms like thermal spike^2–7^, Coulomb explosion^8,9^ and more refined models involving the role of target inner-shell electron excitation^10^ and exciton decay^11^ etc. The damage mechanism leading to the atomic movements in the track region however is still not clear. To alienate one mechanism over the other, Benyagoub^12–14^ studied SHI induced structural phase transformation in ZrO_2_ and HfO_2_, which have similar physical and chemical properties but have important differences like specific gravity and transition temperature from one phase to the other.

The objective of the present study is to impose further constraints on these models by examining the irradiation sensitivity of the two important phases of TiO_2_, namely anatase and rutile, which share the same chemical composition, but have different physical properties. For the application point of view, TiO_2_ is used in photocatalysis, hydrogen production, fuel cells, gas sensors, lithium-ion batteries, super-capacitors, photovoltaic, corrosion protective coatings^15^ and also is a significant chemical component of ceramic nuclear wastes^16^. Understanding of the irradiation sensitivity of this material is therefore an important issue. The effect of 297 and 511 MeV Ni ion irradiation in anatase and rutile pellets were investigated by X-ray diffraction (XRD), Raman and UV-Visible spectroscopy. We showed that the predictions of the inelastic thermal spike model are in close agreement with the experimentally observed damage creation.

## Results and Discussion

### Ion irradiation induced damage cross section from XRD study

**Figure 1:**
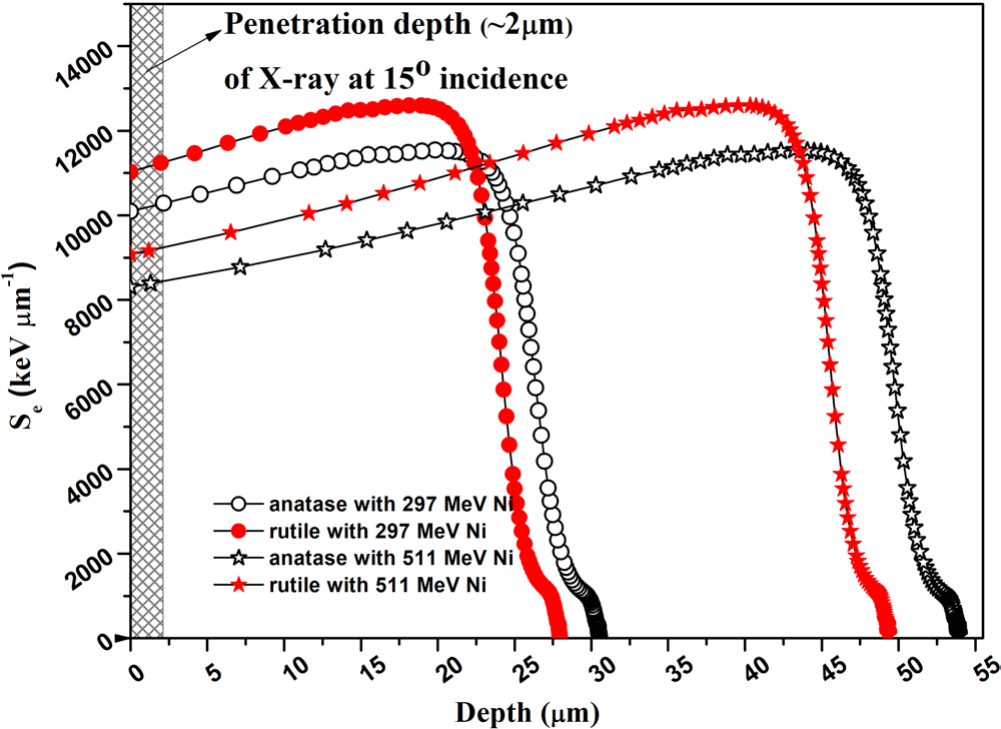
SRIM simulation of electronic energy losses, S_e_ as a function of depth from the surface of TiO_2_(anatase and rutile) pellets irradiated with 297 and 511 MeV Ni ions. The shaded portion represents the region probed by XRD.

The extent of damage created by an energetic ion in a material basically depends on the energy that the ion deposits in the material. We therefore determined the S_e_, S_n_ and the range of 297 and 511 MeV Ni ions in the two different phases of TiO_2_, anatase and rutile using the SRIM (stopping and range of ions in matter) code^17^. The different density of anatase and rutile used in SRIM, dictates the difference of these parameters in these phases. Much larger values of S_e_ than S_n_ (Table 1) indicate that the observed modifications in both rutile and anatase is mostly due to S_e_ of the ions and the effect of S_n_ can be neglected. Variation of S_e_with depth from the sample surface is shown in Fig. 1. This figure clearly shows that Ni ions are implanted much deeper in the TiO_2_ pellets as compared to the X-ray probing depth. This figure also shows that S_e_ is nearly constant in the region probed by XRD.

**Table 1:** Ion track radius obtained from the fluence dependence of XRD peak area, i-TS model and the thickness of the halo region obtained from the fluence dependence of the FWHM of XRD peaks of anatase and rutile phase of TiO_2_ subjected to 297 and 511 MeV Ni ion irradiation.

Ni Ion Energy (MeV)	Phases of TiO_2_ irradiated	Mean S_e_ (keV nm^−1^) in the region (5 μm) probed by XRD	Mean S_n_ (keV nm^−1^) in the region (5 μm) probed by XRD	Track radius, R_a_ (nm) from XRD peak area	Track radius (nm) from i-TS model	Thickness (nm) of the halo from XRD peak FWHM
297	Anatase	10.3	0.008	2.3 ± 0.6	0.9	1.7 ± 0.8
	Rutile	11.2	0.009	1.2 ± 0.2	0.6	1.6 ± 0.7
511	Anatase	8.4	0.005	1.1 ± 0.2	0	5.5 ± 1.5
	Rutile	9.1	0.005	—	0	—

**Figure 2:**
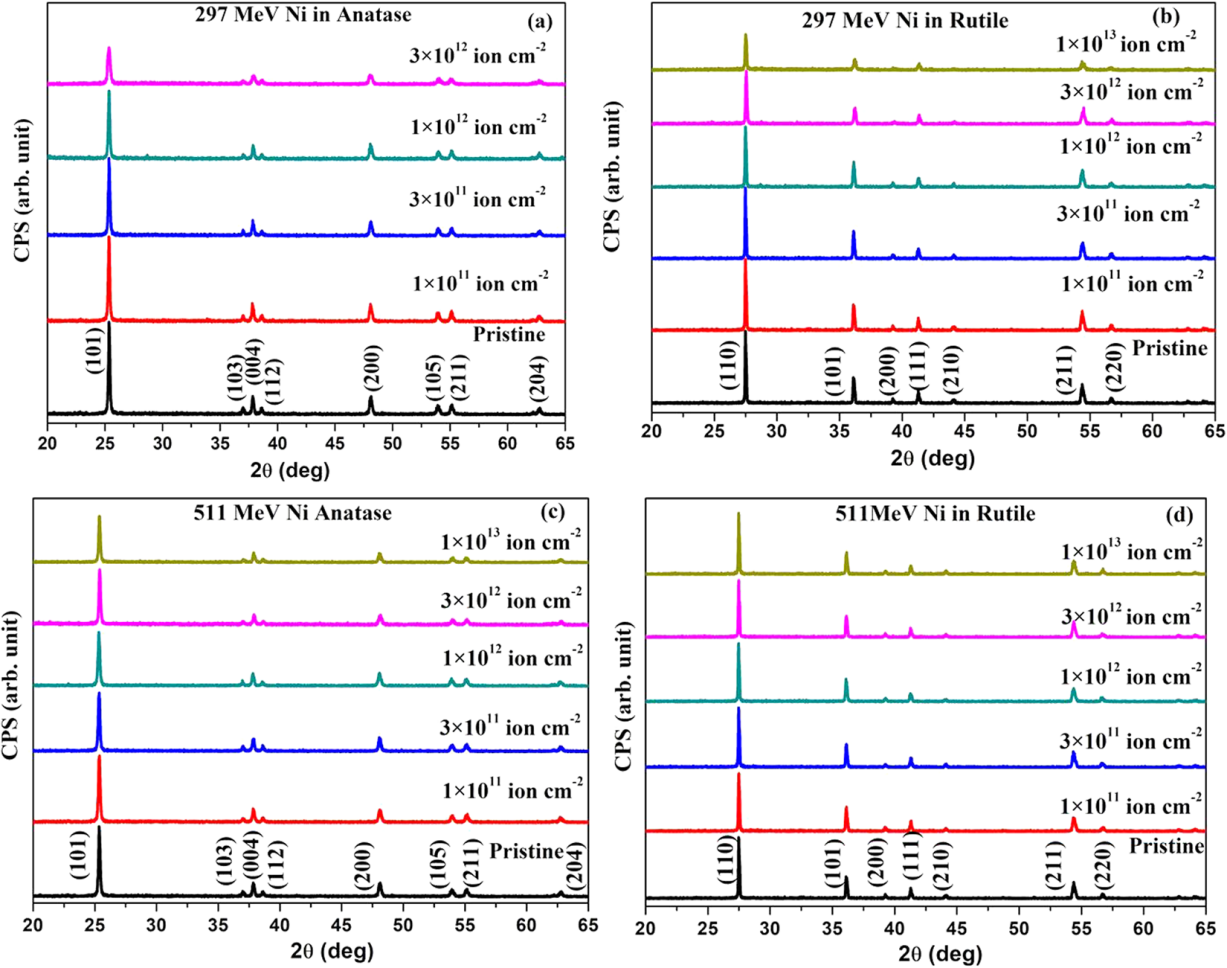
Evolution of the XRD pattern of TiO_2_ with ion fluence. (**a**) Anatase and (**b**) rutile with 297 MeV Ni ion irradiation. (**c**) anatase and (**d**) rutile with 511 MeV Ni ion irradiation.

The XRD patterns before and after irradiation of anatase and rutile phases of TiO_2_ with 297 and 511 MeV Ni ions at different fluences are shown in Fig. 2. Exclusion of the highest intense peak makes the low intense peaks in Fig. 2 clearly visible (Supplementary Fig. S1). The unit cell parameters a = 3.779 Å, c = 9.439 Å for anatase and a = 4.583 Å, c = 2.96 Å for rutile determined from the XRD patterns compare well with the reported values (JCPDS card No. 83–2243, 89–4920). The unit cell parameters did not change and no new peaks appeared in the XRD patterns on irradiation by 297 or 511 MeV Ni ions up to the highest fluence (1 × 10^13^ ions cm^−2^). Irradiation affected only the area and the full width at half maxima (FWHM) of the peaks to some extent. We therefore studied the fluence dependence of these parameters for quantitative estimation of the damage creation under SHI irradiation.

**Figure 3:**
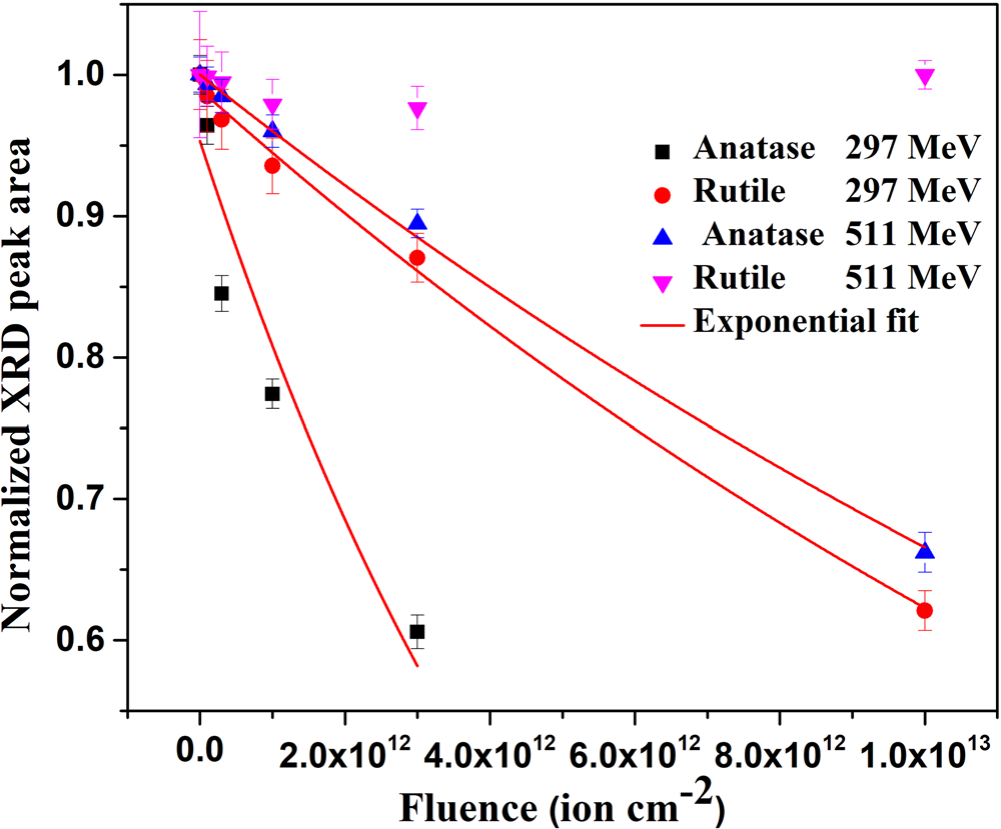
Evolution of area of the most intense peaks of anatase and rutile (both normalized to the area of this peak for the pristine samples) with 297 MeV and 511 MeV Ni ion fluence. Solid lines represent fitting of the experimental plot to the Poisson equation (equation (1)).

A comparison of the damage efficiency of 297 and 511 MeV Ni ions in anatase and rutile phases of TiO_2_ as assessed from the evolution of the area under their most intense XRD peaks with ion fluence is shown in Fig. 3. Data for anatase with 297 MeV Ni ion irradiation at the fluence 1 × 10^13^ ions cm^−2^ is not shown in this figure since this pellet was found broken during dismounting of the samples from the sample holder at the end of the irradiation experiment. Area of the XRD peaks decreased with increasing ion fluence in all cases except in the case of the rutile sample irradiated with 511 MeV Ni ions. Rutile samples were not affected by 511 MeV Ni ion irradiation even up to the highest fluence. Evolution of the area under XRD peaks with 297 and 511 MeV Ni ion fluences thus indicate that anatase is more sensitive to irradiation than the rutile phase of TiO_2_.

The electronic energy loss of 511 MeV Ni ions in rutile as estimated from SRIM code^17^ is 9.1 keV nm^−1^. This observation thus indicates that the S_eth_ for the creation of latent tracks in rutile samples is higher than 9 keV nm^−1^. This value however differs from the S_eth_ (6.2 keV nm^−1^) obtained by Nomura *et al*.^18^, but agrees with the S_eth_ (10 keV nm^−1^) reported by Karlusic *et al*.^19^. Many studies have reported that ion track radius increases and S_eth_ decreases when the irradiation is performed at lower ion velocity^20–22^. We thus presume that higher velocity of the 511 MeV Ni ions compared to that of the low velocity ions used by Nomura *et al*.^18^ justify this deviation.

A comparison of the rate of decrease of the area under the XRD peaks with 297 MeV and 511 MeV (Fig. 3) Ni ion fluence for both anatase and rutile clearly indicates the higher damage efficiency of 297 MeV Ni ions than that of 511 MeV Ni ions. The damage efficiency thus scales with the S_e_ value of the ions, which is higher in the case of 297 MeV Ni ions (Table 1). Also lower velocity of 297 MeV Ni ions (3.12 × 10^9^ cm s^−1^) than that of 511 MeV Ni ions (4.1 × 10^9^ cm s^−1^) is expected to further enhance the damage efficiency of the lower energy ions. Even though samples were progressively damaged with increasing ion fluence, they retained their crystallinity even at the highest fluence of either 297 or 511 MeV Ni ion irradiation (Fig. 2).

The damage cross section was estimated from the fluence dependence of the area of the most intense diffraction peak viz. (101) of anatase and (110) of rutile phases by using the following equation based on Poisson statistical model^23^.

1 $${\rm{A}}({\rm{\phi }}t)={{\rm{A}}}_{\infty }+(1-{{\rm{A}}}_{\infty }){{\rm{e}}}^{-{{\rm{\sigma }}}_{{\rm{a}}}{\rm{\phi }}{\rm{t}}}$$

where A(φt) and A_∞_ are the areas under the XRD peak at an ion fluence (φt) and saturation value of the area at high ion fluences (φt → ∞) respectively, both normalized to the area of the corresponding peak of the pristine sample. The σ_a_ is the damage cross section. Fluence dependent normalized area under the XRD peaks (Fig. 3), fitted to equation (1) yielded the cross section of an ion track. The radii of the ion tracks, R_a_ (assuming cylindrical track geometry) for 297 MeV and 511 MeV Ni ions in anatase and rutile were obtained from the respective cross sections and are given in Table 1 for comparison of the irradiation sensitivity of anatase and rutile with the predictions of different models as discussed latter.

**Figure 4:**
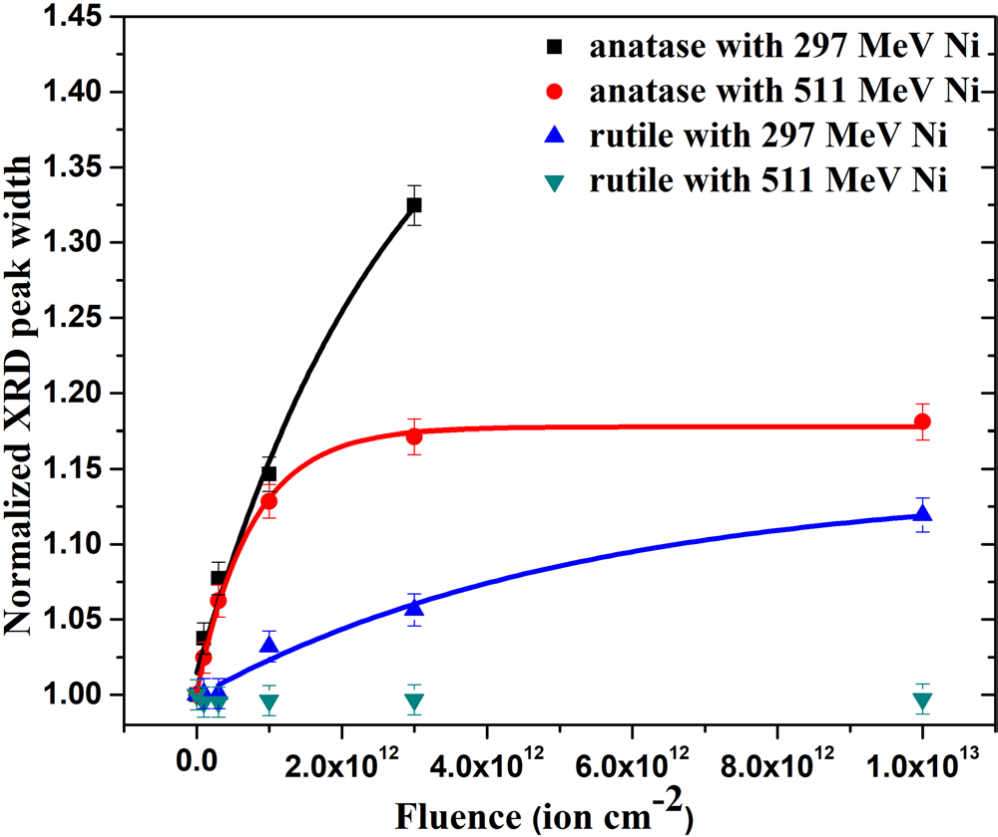
Evolution of FWHM of the most intense XRD peaks of anatase and rutile (normalized with respect to the corresponding pristine sample) with 297 and 511 MeV Ni ion fluence. Solid lines represent fitting of the experimental data to the Poisson equation (equation (2)).

Similarly to the evolution of the area under the peaks, the fluence dependence of the FWHM (Fig. 4) shows higher sensitivity of anatase to irradiation by both 297 and 511 MeV Ni ions than the rutile phase. This figure also shows higher damage efficiency of 297 MeV Ni ions than that of the 511 MeV Ni ions for both anatase and rutile phases. The FWHM of the rutile TiO_2_ XRD peak in fact was not affected by 511 MeV Ni ion irradiation, indicating that the S_eth_of rutile is higher than 9 keV nm^−1^ as was suggested from the fluence dependence of the area under the peak (Fig. 3).

The variations of the normalized FWHM values, W with ion fluence in each case (Fig. 4) was fitted to Poisson equation^4^

2 $${\rm{W}}({\rm{\phi }}{\rm{t}})={{\rm{W}}}_{\infty }\times (1-{{\rm{e}}}^{-{{\rm{\sigma }}}_{{\rm{w}}}{\rm{\phi }}{\rm{t}}})+1$$

where σ_w_ is the cross section of the irradiation induced modified region contributing to the fluence dependence of the FWHM. This modified crystalline region surrounds the amorphous core^24^. (W_∞_+1) is the maximum value of the normalized FWHM for φt→∞. Considering a halo of cross section σ_W_ surrounding the track core of cross section σ_a_, we extracted an equivalent radius R_W_ of the halo region from the relation ${{\rm{\sigma }}}_{{\rm{a}}}+{{\rm{\sigma }}}_{{\rm{w}}}={{\rm{\pi }}{\rm{R}}}_{{\rm{w}}}^{2}$. The thickness of the halo region ${\rm{t}}\,(={{\rm{R}}}_{{\rm{w}}}-{{\rm{R}}}_{{\rm{a}}})$ for all the four cases is given in Table 1. Except for rutile irradiated with 511 MeV Ni ions, where neither the area nor the FWHM of the XRD peaks were affected, all other cases indicate the presence of a halo structure surrounding the heavily damaged core along the ion path. The high resolution transmission electron microscopy (HRTEM) images had also indicated a similar halo like region surrounding the latent tracks in 84.5 MeV Cu ion irradiated rutile TiO_2_^18^. The exact microstructure of the halo region, though is not apparent in the present study, some of the following observations give an indication of the irradiation induced modifications in these regions.

To understand the defect structure in the halo region contributing to the increase of FWHM with ion fluence, we take note of the fact that thickness of the halo region, t is much larger for 511 MeV Ni ion than for 297 MeV Ni ion irradiated anatase, though opposite is the case for R_a_. Thus the thickness of the halo scales with the energy rather than the energy loss of the ions. This observation clearly indicates an effect opposite to the conventionally observed velocity effect^20,21^. Volkov *et al*.^22^ have reported similar observation in LiF. This unusual result seems to be a consequence of the lattice strains due to oxygen vacancies induced over a larger region by higher velocity ions. Effect of irradiation induced oxygen vacancies is also revealed in Raman and UV-Visible spectroscopic studies discussed below.

### Evolution of Raman spectra indicating irradiation induced oxygen loss

**Figure 5:**
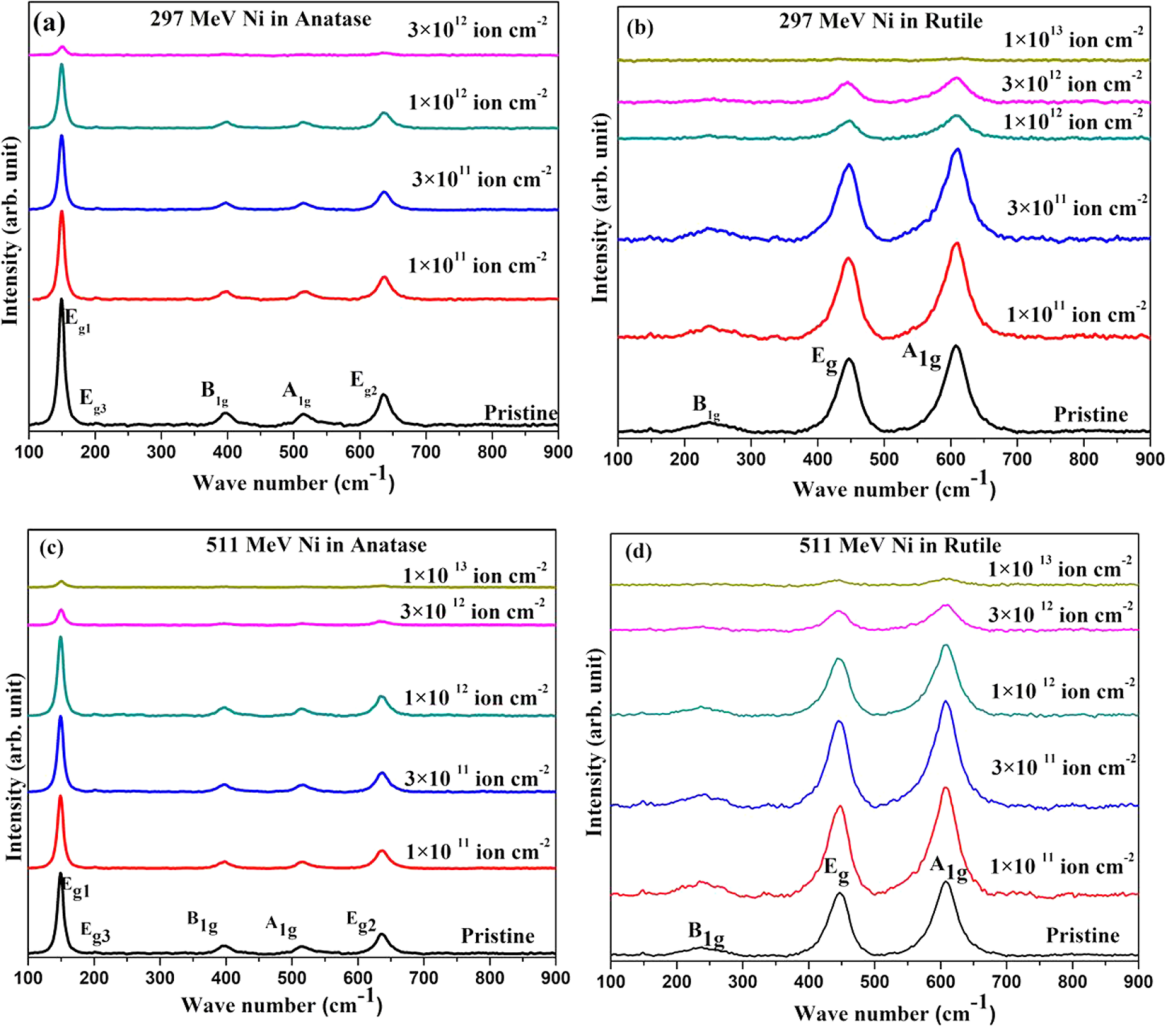
Evolution of the Raman spectra of TiO_2_ with ion fluence. (**a**) Anatase and (**b**) rutile with 297 MeV Ni ion irradiation. (**c**) Anatase and (**d**) rutile with 511 MeV Ni ion irradiation.

The evolution of the Raman spectra of anatase and rutile phases of TiO_2_ with 297 and 511 MeV Ni ion fluences is shown in Fig. 5. In agreement with the reported data^25,26^, the pristine anatase samples show five distinct Raman lines at 149 cm^−1^ (E_g_), 199 cm^−1^ (E_g_), 395 cm^−1^ (B_1g_), 515 cm^−1^ (A_1g_ and B_1g_, unresolved) and 635 cm^−1^ (E_g_) and the pristine rutile samples show three distinct Raman lines at 148 cm^−1^ (B_1g_), 447 cm^−1^ (E_g_) and 608 cm^−1^ (A_1g_). The rutile samples also exhibit a broad peak at 238 cm^−1^ caused by second-order scattering.

Irradiation by 297 MeV and 511 MeV Ni ions strongly suppressed the intensity of the Raman peaks of both anatase and rutile (Fig. 5). Molecular dynamic simulation^27^ indicated a significant irradiation induced distortion of the chemical bonds, caused by the creation of vacant oxygen sites in the TiO_6_ octahedra. This can result into the observed irradiation induced suppression of the Raman peaks. Creation of oxygen vacancies due to irradiation led to darkening of the top layer of the pellets. Laser irradiation has also led to formation black Tisuboxides which are Raman silent^26^. In the present study, the irradiation induced darkened top layer would screen the underlying material and hence would strongly reduce the intensity of Raman peaks with increasing ion fluence as observed. Since suppression of the Raman peaks arises due to irradiation induced oxygen vacancies, these cannot be directly related to the ion tracks. Hence we could not assess the relative sensitivity of the anatase and the rutile phases of TiO_2_ from the fluence dependence of the Raman peaks.

### Irradiation induced modification of the optical band gap

**Figure 6:**
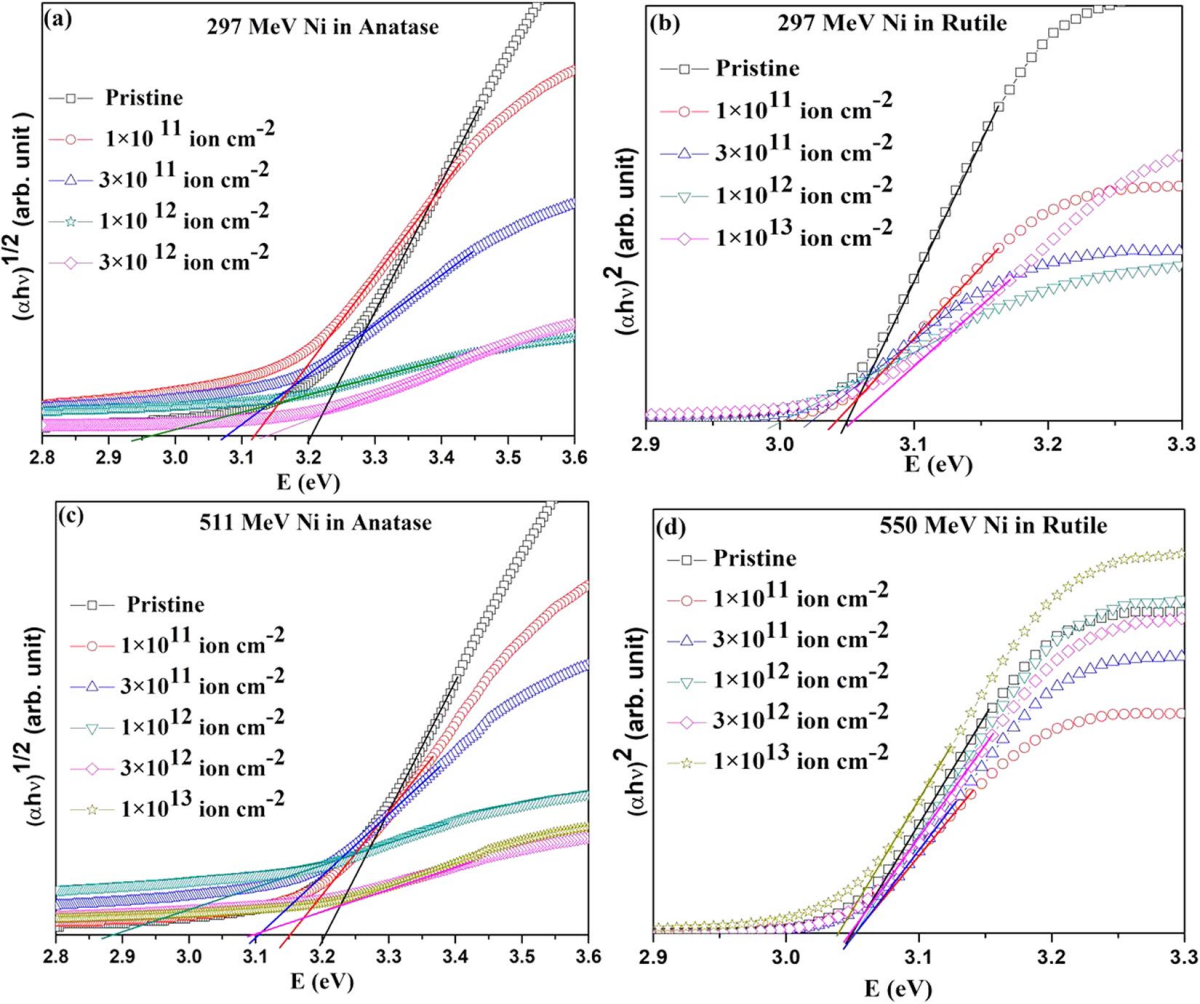
UV–Visible spectra of TiO_2_ at different ion fluences. (**a**) Anatase and (**b**) rutile with 297 MeV Ni ion irradiation. (**c**) Anatase and (**d**) rutile with 511 MeV Ni ion irradiation.

**Figure 7:**
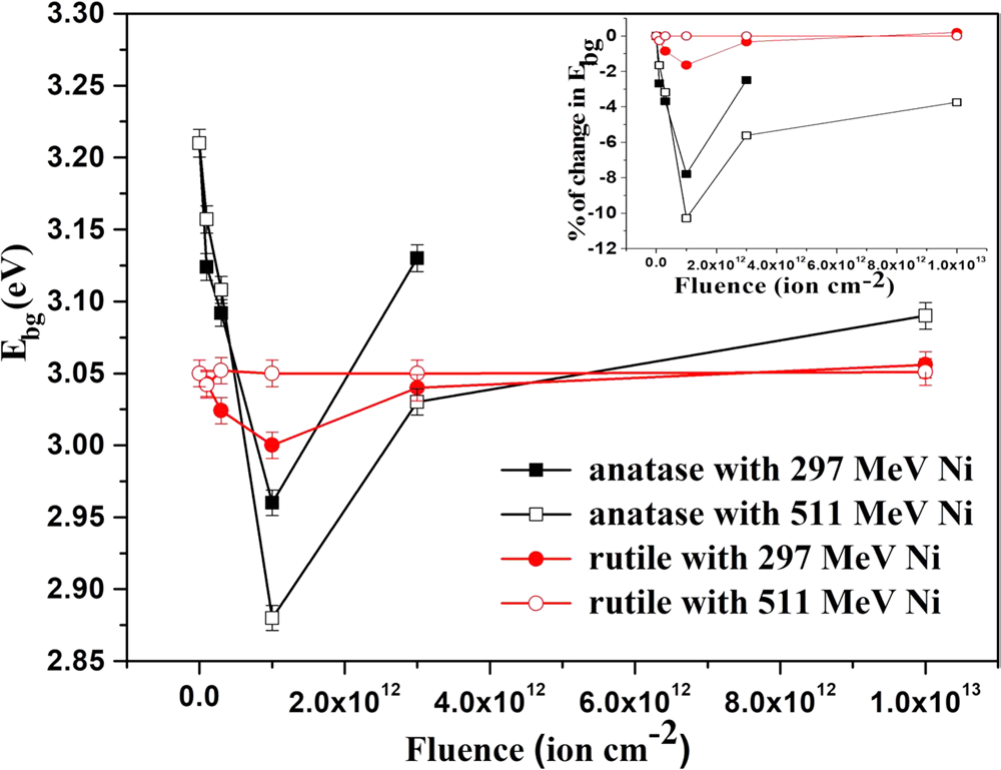
Variation of the optical band gap, E_bg_ of anatase and rutile samples with 297 and 511 MeV Ni ion fluence. Inset shows the corresponding percentage change in E_bg_.

Optical absorption spectra for anatase and rutile pellets irradiated by 297 MeV and 511 MeV Ni ions at different fluences are presented in Fig. 6 as the variation of (αhν)^n^ vs hv using Tauc’s equation^28^

3 $${({\rm{\alpha }}h{\rm{\nu }})}^{n}={\rm{A}}(h{\rm{\nu }}-{{\rm{E}}}_{{\rm{bg}}})$$

where hv is the energy of the incident photons, α is the absorption coefficient, n is the exponent that determines the type of electronic transition causing the absorption (indirect for anatase with $\,n=\frac{1}{2}$ and direct for rutile with *n* = 2)^29^, A is a constant and E_bg_ is the optical band gap. The E_bg_ of anatase and rutile obtained from extrapolation of the curves in Fig. 6 to the energy axis for zero absorption coefficient is plotted as a function of 297 and 511 MeV Ni ion fluences in Fig. 7. The E_bg_ of the un-irradiated anatase and rutile are 3.21 eV and 3.05 eV respectively, which are as reported by others^29^. Irradiation with 297 MeV Ni ions decreased E_bg_ of both anatase and rutile with increasing fluence up to 1 × 10^12^ ions cm^−2^ beyond which, the E_bg_ showed an increase. Irradiation with 511 MeV Ni ions led to a similar variation of E_bg_ with the ion fluence in the case of anatase, but did not affect the E_bg_ of the rutile phase. Figure 7 also shows that the irradiation induced change of E_bg_ in anatase is significantly larger than that in rutile, further confirming the conclusion drawn from the XRD results that the anatase phase is more sensitive to ion irradiation than the rutile phase of TiO_2_.

SHI irradiation induced lattice distortion resulting in the generation of a number of shallow energy levels ordinarily causes decrease in the energy band gap in many systems^30–32^, as is observed in the low fluence regime in the present study. In the low fluence regime, where the highly damaged regions along the ion paths (latent tracks) do not overlap, the rapid decrease of E_bg_ indicates that in addition to the creation of the latent tracks, 297 and 511 MeV Ni ions create lattice distortion due to oxygen loss, as is discussed above, in a region surrounding the tracks.

The decrease of E_bg_ is expected to be higher in the case of the 297 MeV than that of the 511 MeV Ni irradiation due to the higher S_e_ of the former. In anatase TiO_2_, which is modified by both ion beams, the extent of decrease in E_bg_ was found to be opposite to what is expected based on the S_e_ value. We thus found that 297 MeV Ni ion irradiation reduces the band gap of anatase from its pristine value by about 8% at the ion fluence of 1 × 10^12^ ions cm^−2^ while 511 MeV Ni ion irradiation at the same ion fluence, reduces the band gap by more than 10% (Inset of Fig. 7). While the radius, R_a_ extracted from the area under the XRD peaks, scaled with the S_e_ of the ions, the thickness of the halo (t) showed exactly the opposite trend (Table 1), like the energy gap, E_bg_. Both E_bg_ and t scale with the ion energy rather than the energy loss. This unusual result, contradicting the predictions of the velocity effect^20,21^, seems to be a consequence of the lattice strains due to oxygen vacancies in a cylindrical region surrounding the latent tracks^33^, as discussed earlier.

The optical band edge of amorphous TiO_2_ (3.38 eV)^34^ is higher than that of anatase (3.21 eV) and rutile (3.05 eV). With increasing ion fluence, volume fraction of the latent tracks with effective radius R_a_ (Table 1) would dominate over the softly defected regions around these tracks. The system will therefore be driven towards an amorphous state. As a consequence, the band gap would increase in the high fluence regime. Conforming to this expectation, the E_bg_ was found to increase beyond the fluence of 1 × 10^12^ ions cm^−2^ irrespective of the system (anatase or rutile) or the ion beam (297 or 511 MeV Ni ions) used for irradiation (Fig. 7). If the samples were completely amorphized, one would expect higher E_bg_ in irradiated samples than in pristine sample. But it is not the case here, since the crystallinity of the samples is still retained at the highest ion fluence as revealed from the XRD study.

### Irradiation sensitivity of anatase and rutile, and mechanism of track formation

We now examine the applicability of the two generally used models of ion-matter interaction, namely the Coulomb explosion model and the thermal spike model to understand the difference in the sensitivity of TiO_2_ in its anatase and rutile phases to SHI irradiation as indicated from the XRD and optical band gap studies. A few studies^35,36^ have indicated that both the Coulomb explosion and the thermal spike models may be simultaneously operative in the wake of an energetic ion in a medium. The relative contributions of these models to the observed ion induced modifications however, has not yet been fully clarified. The present work is a step in that direction to examine the contributions of these processes to the observed ion induced modifications in the materials by analyzing the details of their predictions based on the physical parameters of the system under consideration.

According to the Coulomb explosion model^8^, the passage of an energetic heavy ion through a material produces a large concentration of positively charged ions due to the escape of the excited electrons out of a cylindrical region surrounding the path of the ion. The created ions repel each other by electrostatic force and explode radially causing lattice disorder along the ion path, called the latent track, provided the electrostatic stress created in the wake of the ion overcomes the local mechanical (or bonding) strength, thus satisfying the following equation^8^.

4 $${{\rm{\eta }}}^{2} > {\rm{R}}\equiv \frac{{\rm{Y}}{\rm{\epsilon }}{{\rm{a}}}_{0}^{4}}{10{{\rm{e}}}^{2}}$$

where *η* is the average ionization, R is the stress ratio, ∈ is the dielectric constant, a_0_ is the average atomic spacing, e is the electron charge and Y is the Young’s modulus. The parameter ‘R’ is used to calculate the relative sensitivity of various materials to the formation of ion tracks^8^.

Equation (4) predicts that track formation is easier in anatase due to its lower values of Y, ∈ and a_0_ than in rutile (Table 2). Evolution of the XRD peak area (Fig. 3), FWHM of the peaks (Fig. 4) and optical band gap (Fig. 7) of the anatase and rutile pellets irradiated with both 297 MeV and 511 MeV Ni ions qualitatively agree with the prediction of the Coulomb explosion model.

**Table 2:** Physical parameters of anatase and rutile TiO_2_.

Parameters	Anatase	Rutile
Average Young’s modulus (GPa)^46^	211	350
Average Dielectric constant^46^	37	117
Average atomic spacing (Å)^46^	2.48	2.56
Stress Ratio ‘R’ as per Coulomb Explosion Model	12.8	76.2
Solid density (gm cm^−3^)^46^	3.89	4.25
Liquid density (gm cm^−3^)^46^	2.4	2.4
Specific heat capacity (J K^−1^ gm^−1^)^46^	0.93	0.93
Melting temperature, T_m_ (K)^46^	1949	2130
Thermal conductivity (J K^−1^ cm^−1^ s^−1^)^46^		
>300 K	0.084	0.084
1500 K	0.03	0.03
Latent heat of fusion (J gm^−1^)^46^	726.05	838.16
Vaporization temperature (K)^46^	3200	3200
Latent heat of vaporization (J gm^−1^)^46^	6157	6157
Carrier density, (N per cm^−3^)^47^	2 × 10^19^	2.5 × 10^19^
Electron effective mass, m* in units of free electron mass^47^	1	20
Linear absorption coefficient, κ (μm^−1^)^48^	0.0503	0.0549

**Figure 8:**
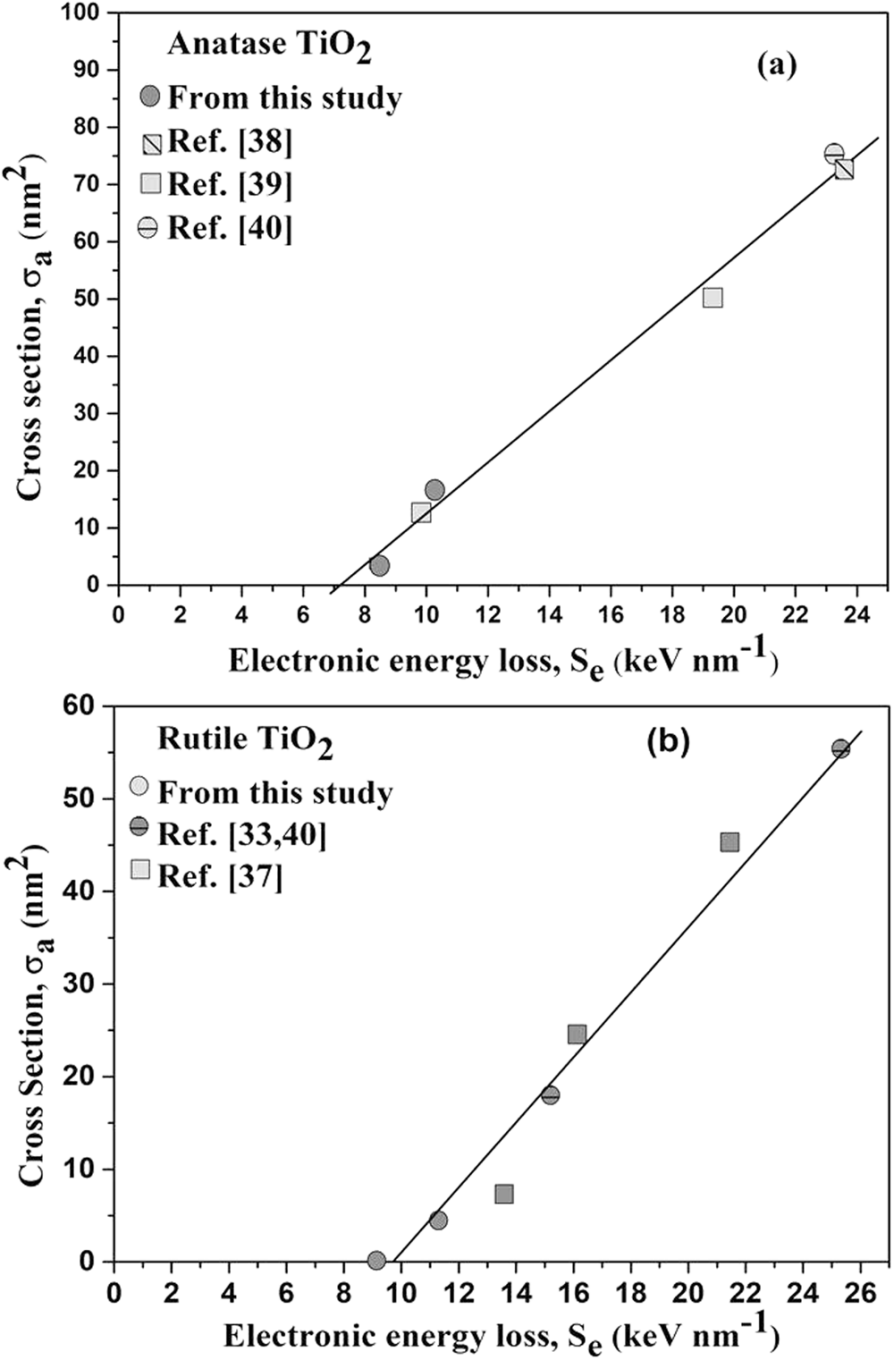
Evolution of the cross section σ of ion tracks in (**a**) anatase and (**b**) rutile with electronic energy loss using the data of the present study as well as studies reported earlier. Extrapolation to null damage cross-section gives an electronic stopping power threshold of damage creation.

To make a quantitative comparison, we present the evolution of the cross section of ion tracks, σ_a_ with electronic energy loss, S_e_ for these phases (Fig. 8) using the data obtained from the present study (Table 1) and from studies reported in the literature^33,37–40^. In contrast to the prediction of the Coulomb explosion model^9^ that, σ_a_ should scale as ${{\rm{S}}}_{{\rm{e}}}^{4}$, the data presented in Fig. 8 show linear variation of the σ_a_ with S_e_. In addition the S_eth_ values (7.1 ± 0.8 keV nm^−1^ for anatase and 9.9 ± 1.3 keV nm^−1^ for rutile) estimated by extrapolating the σ_a_ vs S_e_ to null damage cross-section in Fig. 8, do not agree with the predictions of Coulomb explosion model as discussed below.

A refinement of the Coulomb explosion model^9,41^ indicates that the radial recoil energy, E_r_ transmitted by the incident ion to the lattice atoms surrounding the ion path, leading to the formation of tracks in a solid, is proportional to ${{\rm{S}}}_{{\rm{eth}}}^{4}/{{\rm{\rho }}{\rm{\omega }}}_{{\rm{p}}}^{2}$, where ρ is the density of the target and ω_p_ the electron plasma frequency. The latter is estimated using the formula ${{\rm{\omega }}}_{{\rm{p}}}^{2}=\frac{{{\rm{Ne}}}^{2}}{{{\rm{\varepsilon }}}_{0}{{\rm{m}}}^{\ast }}$, where ε_0_ is the permittivity of free space, e is the electron charge, N is the carrier density and m* is the effective electron mass of the material. Using the values of ε_0_ and e along with the values of ρ, N and m* (Table 2), the ratio, $\frac{{({{\rm{S}}}_{{\rm{eth}}})}_{{\rm{rutile}}}}{{({{\rm{S}}}_{{\rm{eth}}})}_{{\rm{anatase}}}}$ is found to be 0.51. Coulomb explosion thus predicts S_eth_ of rutile is almost half of the S_eth_ of anatase. On the contrary, the experimental data (Fig. 8) shows the S_eth_ of rutile (9.9 keVnm^−1^) is larger than that of the anatase phase (7.1 keV nm^−1^) of TiO_2_.

The Coulomb explosion model therefore fails to explain the observed variation of the cross section of ion tracks, σ_a_ with electronic energy loss, S_e_ as well as the relative magnitudes of S_eth_ of the two phases of TiO_2_.

Contrary to the Coulomb explosion model, the thermal spike model^2–7^ envisages that a fraction of the energy deposited by SHI to the target electrons is transferred to the lattice atoms, which heats up the lattice in a confined region along the ion path within a short duration, the so called thermal spike. Track forms due to quenching of the region melted due to the spike. Presently, two versions of the thermal spike model are proposed to explain track formation in solids, namely the inelastic thermal spike (i-TS)^3–5^ and the analytical thermal spike (a-TS)^6,7^ models. We first examine the applicability of a-TS model to explain the different irradiation sensitivity of anatase and rutile phases of TiO_2_ as observed in the present study.

The a-TS version of the thermal spike^6,7^ is based on the assumption that soon after the passage of the irradiating ion, a fraction g of the electronic energy loss S_e_ is converted to heat for the atomic lattice. The consequent lattice temperature has a Gaussian distribution with an initial radial extension, a(0), which evolves with time. According to this model, the S_eth_ is obtained by the following equations^7^.

5 $${{\rm{S}}}_{{\rm{eth}}}={{\rm{\pi }}{\rm{a}}}^{2}(0){\rm{\rho }}{\rm{c}}({{\rm{T}}}_{{\rm{m}}}-{{\rm{T}}}_{{\rm{ir}}})/{\rm{g}}$$

where ρ, c and T_m_ are the density, specific heat and melting temperature of the irradiated material, and T_ir_ is the irradiation temperature (300 K in the present case). In this model, the efficiency g has close values in various insulators^6^ and hence is expected to be same for anatase and rutile TiO_2_. The magnitude of g however depends on the ion energy depicting the velocity effect. In the extreme limits of ion energies, g takes the value 0.4 for low energy ions (E < 2 MeV/amu) and 0.17 for high energy ions (E < 7 MeV/amu)^6^. We have thus taken g = 0.17 for 511 MeV Ni case, which corresponds to E = 8.8 MeV/amu. For 297 MeV Ni ion, the E(=5.1 MeV/amu) takes a value between the low and the high energy limits. For this case we have used Fig. 3 of Szenes *et al*.^6^, which shows the variation of g with the E and gives g ∼ 0.2 for 5.1 MeV/amu corresponding to 297 MeV Ni ions. The initial radial extension a(0) is 4.5 nm for 0.1 < E < 20 MeV/amu for all insulators without exception^6^. The a-TS model thus envisages that the S_eth_ for a material would basically depend on the magnitude of its thermo-physical parameters ρ, c and T_m_ (Table 2) in addition to depending on the value of g (velocity effect). The lower values of ρ and T_m_ of anatase than that of rutile, and similar values of other parameters (Table 2) indicate higher sensitivity of anatase to SHI irradiation than rutile as is observed in the present study.

To make a quantitative comparison, we estimate from equation (5), the value of S_eth_ of anatase and rutile using the value of g and other parameters (Table 2) as 11.8 and 14.4 keV nm^−1^ respectively in case of 297 MeV Ni ion. The corresponding values of S_eth_ in case of 511 MeV Ni ions are 13.9 and 16.9 keV nm^−1^. These values being larger than the S_e_ (Table 1) of both 297 and 511 MeV Ni ions in TiO_2_, clearly indicate that these ions cannot create latent tracks in either anatase or rutile. This is in contrast to the experimental observation in the present study, which shows that 297 MeV Ni ions indeed create tracks in both anatase and rutile; and 511 MeV Ni ions create tracks in anatase phase of TiO_2_. We thus find that the a-TS model is not applicable to the present case.

**Figure 9:**
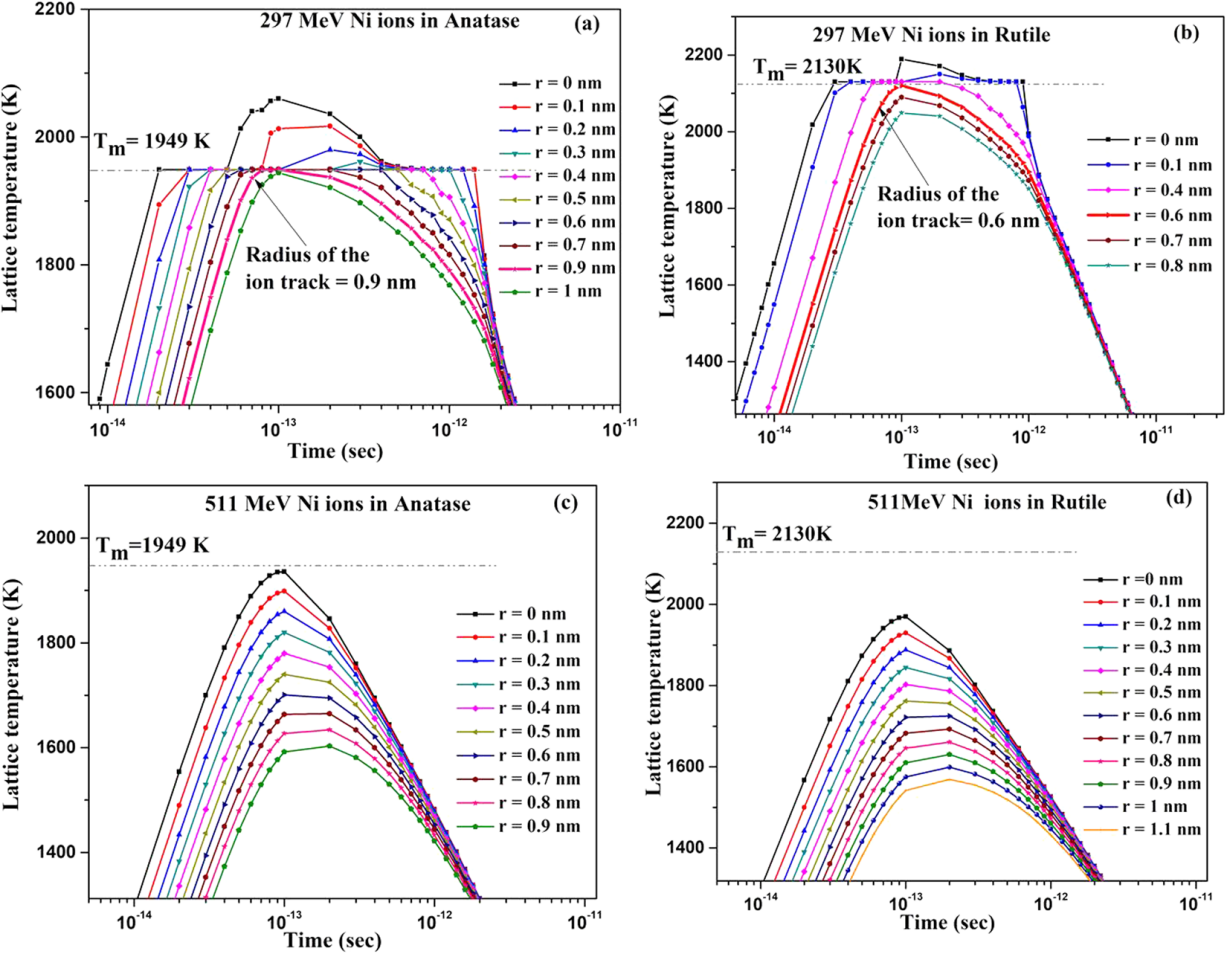
The time evolution of the lattice temperature at several radii from the ion path in (**a**) anatase (**b**) rutile irradiated with 297 MeV Ni ion and (**c**) anatase (**d**) rutile irradiated with 511 MeV Ni ion.

The i-TS model^3–5^ envisages local thermalization in the electronic system due to electron–electron interaction in about 10^−15^ s after the passage of the irradiating ion. Energy locked to the electrons is then transferred to the lattice by electron-phonon coupling in the time scale of 10^−14^ and 10^−12^ sec leading to a large increase in lattice temperature and producing a thermal spike. This model involves numerical solution of the two coupled differential equations (Eqs (7 and 8) in ref.^3^) governing heat diffusion in both electrons and the lattice subsystems in a cylindrical geometry. Besides the thermal data of the irradiated materials, this model needs the knowledge of the electron–phonon mean free path, λ, which is linked to the band gap energy in insulators^5,37^ and found to be 5.8 nm TiO_2_. Using this λ value and the values of the other thermo-physical parameters (Table 2), the time-evolution curves of lattice temperatures for 297 and 511 MeV Ni ion irradiation in anatase and rutile phase of TiO_2_ were simulated (Fig. 9a–d).

As shown in these simulated curves, each 297 MeV Ni ion melts anatase within a radius of 0.9 nm, while it does so within a radius of 0.6 nm in rutile. Quenching of the spike freezes the melted zones leading to formation of ion tracks. For 511 MeV Ni ion, the simulation however shows that in case of anatase, the peak temperature just touches its melting temperature (Fig. 9c), whereas it is far below the melting temperature for rutile (Fig. 9d), thus precluding the formation of tracks in this case. These simulated results based on i-TS model thus establish higher sensitivity of anatase to SHI irradiation than rutile and hence agree with our experimental observation. There is, however, a deviation in the magnitude of the track radius obtained through simulation based on i-TS model and that observed experimentally (Table 1). This mismatch in the values of simulated and experimentally obtained track radius could possibly be a consequence of factors discussed below.

Using high resolution electron microscopy Houpert *et al*.^42^ have shown that the damage cross section having radius less than 2.5 nm are in the form of extended damage fragments in magnetic insulators, ensuing discontinuous ion track. Such discontinuous tracks have also been seen in rutile TiO_2_^19,43^. The radius of the ion tracks observed by us in all the cases is less than 2.5 nm (Table 1). Therefore, the tracks of 297 MeV and 511 MeV Ni ions in anatase and rutile TiO_2_ may not be continuous.

The criterion for amorphization of an insulator under SHI irradiation has been shown to be directly linked to the strength of the ionic bonding^4^. Larger ionicity makes the insulator less prone to amorphization. The bond-type criterion given by Matzke *et al*.^44^ in fact indicate that if the ionicity of the material is more than 60%, it is not amorphized under ion irradiation. Interestingly, the ionicity of TiO_2_ is ∼60% and hence is at the border line of amorphizable and non-amorphizable insulators. Therefore the tracks due to 297 MeV and 511 MeV Ni ions can create high lattice damage instead of completely amorphizing the materials along the ion path.

To conclude, our studies established higher sensitivity of anatase phase than the rutile phase of TiO_2_ to SHI irradiation. The relative sensitivity of anatase and rutile to SHI irradiation qualitatively agreed with the predictions of the Coulomb explosion and the two versions of the thermal spike models. A detailed analysis however, indicated that the predictions of the Coulomb explosion and the analytical thermal spike models do not conform to our experimental observation. The inelastic thermal spike model agreed with our observation albeit with a difference in the radius of ion tracks. Our XRD, Raman spectroscopy and optical band gap studies indicate that SHI irradiation also causes oxygen loss and induces significant lattice distortion in a region surrounding the ion path. As is expected, the radius of the amorphized latent tracks scaled with the electronic energy loss of the ions. But the radius of the softly defected crystalline region surrounding the ion track was found to scale with the velocity of the ions, while the established velocity effect predicts the opposite variation.

## Methods

Polycrystalline anatase and rutile pellets of 10 mm diameter and 1 mm thickness were prepared from commercial TiO_2_ powder (Merck, 99% purity) in anatase form at a pressure of 1000 kg cm^−2^. A set of pellets was sintered at 873 K and another set was sintered at 1323 K for 1 hour. Sintering improved mechanical strength of the pellets for easy handling during irradiation and characterization. Sintering at 873 K retained the anatase phase and at 1323 K converted anatase to rutile phase.

The samples were however rather brittle as they could crumble under scratching. Therefore, to prevent them from shattering under the ion beam, they were covered with aluminum foils and then firmly attached with specific frames to improve their mechanical, electrical and thermal contact with the sample holder. Two thicknesses of aluminum foils were chosen: 20 and 55 μm. The ion irradiations of the anatase and rutile pellets were performed at room temperature and under normal incidence at the medium energy beam facility of the GANIL accelerator in Caen (France). As the energy of the ^58^Ni^24+^ ions delivered by the accelerator was 617 MeV, according to the SRIM code^17^ these ions traversed the aluminum sheets and entered the samples with an average energy of 511 MeV in the case of the 20 μm Al foils and with an average energy of 297 MeV in that of 55 μm. For homogeneous irradiation, the beam was scanned over an area of 44 × 44 mm^2^, thus covering the complete surface of the pellets. The irradiation fluences ranged from 1 × 10^11^ to 1 × 10^13^ ions cm^−2^ and to minimize target heating all irradiations were performed with an ion flux limited to 2 × 10^8^ ions cm^−2^s^−1^.

The pristine and the irradiated anatase and rutile pellets were studied by XRD, Micro-Raman and UV-Visible spectroscopy. XRD measurements were done using Bruker D8 advanced diffractometer with Cu-k_α_radiation. Raman spectra were taken using Enwave Optronics Raman spectrometer (EZRAMAN-M) under the excitation by 785 nm laser. UV-visible diffuse reflectance spectra of the samples were recorded in the range 200–900 nm wavelength using Shimadzu UV–Visible double beam spectrophotometer (UV-2450) using BasiO_4_ as a standard. This equipment is equipped with integrating sphere arrangement. The diffuse reflectance spectra were converted to absorbance spectra by the Kubelka-Munk method.

XRD patterns were recorded over the range of 2θ = 20 to 80 degree in steps of 0.01 degree at scan speed of 1 degree per minute. The X- rays were incident at an angle ϕ(=15°) relative to the surface of the pellets to probe a layer of thickness d. After traveling through this layer, the incident X-ray intensity, I_0_ is reduced to ${{\rm{I}}}_{{\rm{d}}}={{\rm{I}}}_{0}{{\rm{e}}}^{-{\rm{\kappa }}{\rm{d}}(\frac{1}{\sin ({\rm{\varphi }})})}$ following the Beer–Lambert law^45^. Here κ is the linear attenuation coefficient of the X-rays through the material. The diffracted X-rays corresponding to a selected 2θ_hkl_ Bragg reflection, after traveling through this layer on its exit, is also reduced by ${{\rm{e}}}^{-{\rm{\kappa }}{\rm{d}}(\frac{1}{\sin (2{{\rm{\theta }}}_{{\rm{hkl}}}-{\rm{\varphi }})})}$. As a result, the total attenuation for X-ray diffracting from depth d is ${{\rm{e}}}^{-{\rm{\kappa }}{\rm{d}}(\frac{1}{\sin ({\rm{\varphi }})}+\frac{1}{\sin (2{{\rm{\theta }}}_{{\rm{hkl}}}-{\rm{\varphi }})})}$. Therefore, with the attenuation of the X-ray limited to e^−1^, the probed depth is ${\rm{d}}=\frac{1}{{\rm{\kappa }}(\frac{1}{\sin ({\rm{\varphi }})}+\frac{1}{\sin (2{{\rm{\theta }}}_{{\rm{hkl}}}-{\rm{\varphi }})})}$. With 2θ_hkl_ value corresponding to the most intense XRD peak, ϕ(=15°) and the value of κ for anatase and rutile (Table 2), the depth probed by the XRD is 2.10 ± 0.04 μm and 2.14 ± 0.04 μm respectively for these phases. These values are well within the range of the ions in the pellets (Fig. 1) and hence X-rays probe only the region modified by SHI irradiation.

### Data availability

All data generated or analysed during this study are included in this article.

## Electronic supplementary material

Supplementary Information
