# Supplementary material for: Sensitivity of Anatase and Rutile Phases of TiO2 to ion irradiation: Examination of the applicability of Coulomb Explosion and Thermal Spike Models
Source: Sci Rep. 2018 Aug 6;8:11774. doi: 10.1038/s41598-018-30281-4 (PMC6079046; doi:10.1038/s41598-018-30281-4)
Supplement: Supplementary file 1 — Supplementary Information [file 41598_2018_30281_MOESM1_ESM.pdf]

Sensitivity of Anatase and Rutile Phases of TiO_2 to ion irradiation: Examination of the applicability of Coulomb Explosion and Thermal Spike Models

Haripriya Rath[1, 2], B.N. Dash[1, 3], A. Benyagoub[4] & N.C. Mishra[1*]

[1]*Department of Physics, Utkal University, Bhubaneswar, 751004* Odisha, *India*

[2]*Institute of Physics, Sachivalaya Marg, Bhubaneswar, 751005,* Odisha, *India*

[3]*Department of Physics, Salipur College, Salipur, 754103,* Odisha, *India*

[4]*CIMAP (ex-CIRIL-GANIL), CEA-CNRS-ENSICAEN-Université de Caen, F-14070 Caen Cedex, France*

* Correspondence and requests for materials should be addressed to N.C.M

(Email*: nareshcmishra@gmail.com)*

Supplementary Figure

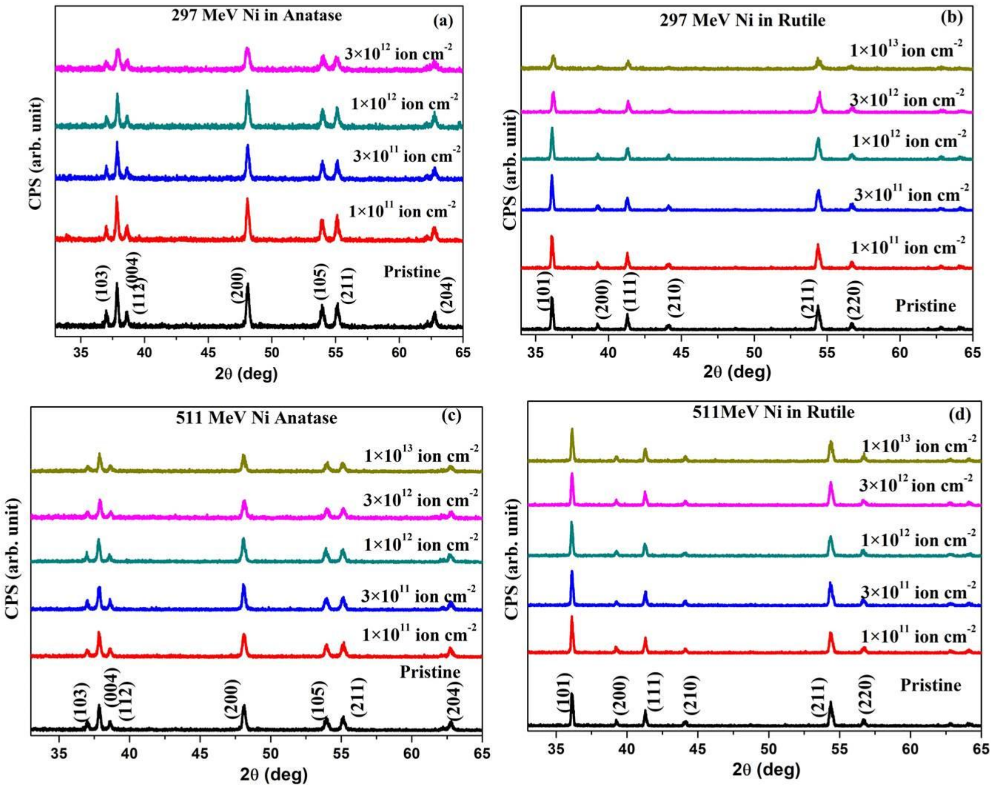

Figure S1. Evolution of the XRD pattern (low intense XRD peaks) of TiO_2 with ion fluence. (a) anatase and (b) rutile with 297 MeV Ni ion irradiation. (c) anatase and (d) rutile with 511 MeV Ni ion irradiation.
